# Polygenic risk, population structure and ongoing difficulties with race in human genetics

**DOI:** 10.1098/rstb.2020.0427

**Published:** 2022-06-06

**Authors:** Jonathan Michael Kaplan, Stephanie M. Fullerton

**Affiliations:** ^1^ Philosophy Program, Oregon State University, Corvallis, OR 97331, USA; ^2^ Department of Bioethics and Humanities, University of Washington School of Medicine, Seattle, WA 98195, USA

**Keywords:** clinical translation, genetic variation, health disparities, polygenic risk score, population structure, race

## Abstract

‘The Apportionment of Human Diversity’ stands as a noteworthy intervention, both for the field of human population genetics as well as in the annals of public communication of science. Despite the widespread uptake of Lewontin's conclusion that racial classification is of ‘virtually no genetic or taxonomic significance’, the biomedical research community continues to grapple with whether and how best to account for race in its work. Nowhere is this struggle more apparent than in the latest attempts to translate genetic associations with complex disease risk to clinical use in the form of polygenic risk scores, or PRS. In this perspective piece, we trace current challenges surrounding the appropriate development and clinical application of PRS in diverse patient cohorts to ongoing difficulties deciding which facets of population structure matter, and for what reasons, to human health. Despite numerous analytical innovations, there are reasons that emerge from Lewontin's work to remain sceptical that accounting for population structure in the context of polygenic risk estimation will allow us to more effectively identify and intervene on the significant health disparities which plague marginalized populations around the world.

This article is part of the theme issue ‘Celebrating 50 years since Lewontin's apportionment of human diversity’.

## 1. Introduction

Richard Lewontin's ‘The Apportionment of Human Diversity’ [1] stands as a noteworthy intervention, both for the field of human population genetics as well as in the annals of public communication of science (for more about the history and impact of this paper, see [2,3]). Though drawing on information about human genetic variation that was both incomplete and crude by contemporary standards, much subsequent research has generally supported the paper's main scientific conclusions [4–6]. Specifically, it is now widely accepted that most of the genetic diversity in the human species exists between individuals within populations and that only a small fraction of the total genetic diversity is accounted for by variation between populations. Lewontin's broader conclusion, that these features of human genetic variation meant that racial classification was of ‘virtually no genetic or taxonomic significance’ and hence should be abandoned for both scientific and sociopolitical use, was left under-developed and has held considerably less sway. Widespread social inequity, rooted in centuries-old beliefs about racial biological distinction and superiority, persists in many parts of the world [7].

Similarly, biomedical research aimed at disentangling genetic and non-genetic contributions to population-level health disparities also continues to grapple with whether and how best to account for racial classification in its work. Nowhere is the latter struggle more apparent than in the latest attempts to translate genetic associations with complex disease risk to clinical use: the polygenic risk score, or polygenic risk scores (PRS). The hope is that PRS can be used to triage high-risk patients for intervention of preventable health problems. But such methods must account for human population structure (the fact that alleles at a locus are not distributed at random across the human species but are more common in people from some locations than others). While PRS attempt to deal with population structure by using principal components of genetic diversity in tests of association with disease, risk estimates are usually applied to patients stratified by self-identified race and/or ethnicity, with a range of consequences arising from the elision of genetic ancestry and social identity. In this paper, we offer some reflections on our ongoing inability to reconcile these complexities of human population structure and discuss the potential implications for clinical use of genetic information in the context of PRS.

## 2. Race(ist) science and Lewontin's ‘Apportionment of Human Diversity’

‘The Apportionment of Human Diversity’ was situated at a particular moment in the history of population genetic investigation and in the face of historical and philosophical recognition of the role that anthropology and biomedicine played in legitimizing claims of racial distinction. Far from being a value-neutral description of population difference, research on the creation of the modern ‘race’ concept has highlighted repeatedly the ways in which it was, from the start, used as a way to justify (for example) colonialism, chattel slavery and genocide as well as other, similarly exclusionary and frankly monstrous, practices (e.g. [8,9]). This kind of work, i.e. ‘race science’ being used to justify social and economic arrangements that perpetuate white superiority, continues to this day [10], and this (mis)use of science had long been a target of Lewontin's analysis and critique [11].

This context explains the move between Lewontin's technical (population genetic) observations and his sociopolitical conclusion. Lewontin noted that of the (roughly) 15% of human genetic diversity that is not attributable to differences between individuals within populations, the larger part of that 15% (approximately 9%) was accounted for by differences between populations *within* ‘conventional racial’ groups (which he identified by reference to a mix of cultural, linguistic and historical information, as well as ‘obvious total genetic divergence’). Only 6% of human genetic diversity appeared to distinguish the seven human races he defined. From this estimate, Lewontin concluded that ‘our perception of relatively large differences between human races and subgroups, as compared to the variation within these groups, is indeed a biased perception and that, based on randomly chosen genetic differences, human races and populations are remarkably similar to each other’ [1, p. 397]. Lewontin went on to end his short article with the matter-of-fact assertion:

Human racial classification is of no social value and is positively destructive of social and human relations. Since such racial classification is now seen to be of virtually no genetic or taxonomic significance either, no justification can be offered for its continuance [1, p. 397].

The point here seemed to be that the very small amount of human genetic diversity for which ‘racial’ classifications accounted was not significant enough to warrant either our usual racial ascriptions or treating racial categories as especially meaningful objects of biological study. In a subsequent publication, Lewontin made this point more explicitly:

The taxonomic division of the human species into races places a completely disproportionate emphasis on a very small fraction of the total of human diversity. That scientists as well as nonscientists nevertheless continue to emphasize these genetically minor differences and find new ‘scientific’ justifications for doing so is an indication of the power of socioeconomically based ideology over the supposed objectivity of knowledge [12, p. 156].

The technical result, and the sociopolitical implication that Lewontin drew from it, became part of the mainstream position that ‘race’ is not a legitimate biological category. So for example, authors such as Biondi & Rickards [13] argue that race is an ‘oversimplification’ and that trying to use the race concept in a biologically meaningful way is ‘a futile exercise’. Race, they argue, does not and cannot adequately capture extant population structure, phylogenetic history, or ‘ecological’ differences. In making this case, they take Lewontin's technical points as one of the key arguments in favour of abandoning the race concept.

On the other hand, others have resisted (and, in some cases, continue to resist) the conclusion that biology cannot support the conception of race demanded by folk racial categories and usage and that race is therefore of ‘virtually no taxonomic significance’. In an article with the subtitle ‘Lewontin's Fallacy’ [14], Edwards argued that the small fraction of the genetic variation accounted for by between-population differences is sufficient to produce robust structuring and that the resulting population genetic clusters can be called ‘races’ without straining the meaning of the term. Other investigators, such as Risch and colleagues [15], with an interest in identifying genetic contributions to complex disease risk, agree and moreover claim that it is precisely those genetic risk factors that differ between races that might help address racial disparities in health outcomes. These authors take issue with Lewontin's claim regarding the non-significance of racial classification, asserting that even if small, the fraction of human genetic variation that differs between races is both relevant and worthy of ongoing investigation.

Irrespective of one's beliefs about the role that genes may or may not play in population health disparities (discussed further in the next section), questions about the scale and biomedical relevance of population genetic differences have continued to matter in genetic investigation. This is on account of the move away from linkage studies in families to investigations of genome-wide association in many thousands of cases and controls, where statistical confounding due to population stratification becomes a potential concern. Of course, the traditional epidemiological approach of matching cases and controls with respect to self-identified race and/or ethnicity (on an assumption of shared genetic background such that confounding can be minimized) has given way to approaches that instead consider genetic ancestry directly. Nevertheless, even with direct incorporation of principal components of genetic variation into association models, it is still the norm to conduct genome-wide association studies (GWASs) in ancestry subgroups and so to estimate effect sizes of gene–disease associations with respect to specific ancestral genetic backgrounds. The ensuing associations, when combined across thousands of loci in the form of a PRS, are tidy but not obviously translatable to patients who self-identify with (or are ascribed to) racial and/or ethnic categories rather than genetic ancestries.

## 3. Racial health disparities and the role of genetic versus social determinants

A major rationale for continuing to study human populations subdivided with regard to racial classification is the observation that many common complex traits and diseases differ in their prevalence between racial and/or ethnic groups, particularly in the United States [16,17]. The existence of pronounced racial and ethnic health disparities, manifest most recently in the stark differences in COVID-19-related hospitalizations and deaths experienced by historically marginalized non-White racial and ethnic groups [18], is a profound public health problem and one that demands urgent redress. It should come as no surprise, therefore, that geneticists eager to do their part to address these disparities would turn their attention to the identification of gene variants that both increase disease risk and are more common in the racial groups burdened by excess disease. In other words, to emphasize precisely that small proportion of the total genetic diversity that Lewontin regarded as of little-to-no significance. Once potential genetic contributors to disparities can be identified, so the reasoning goes, then potent new biological pathways and targets for (likely pharmaceutical) intervention can be devised [19–21].

The difficulty with the pursuit of such research is twofold. First, despite a great deal of effort, relatively few plausible racially stratified gene variants with discernible effects on common complex disease risk have thus far been identified. The most prominent examples in this category include the 8q24 association with prostate cancer risk [22] and the *APOL1* association with end-stage kidney disease risk [23,24]. Second, an abundance of demographic, socioeconomic, environmental and related data affirm the primary role of systemic racism, myriad forms of marginalization and poverty in unequal health outcomes [25–28]. Not only does an overfocus on genetic contributions to racial health disparities distract public policymakers from attending to more relevant drivers of health and disease, it also undermines investigation of the ways in which racial ascription and racism (and the associated stresses of living in a racist society) lead to ill health [29,30].

There is really no debate about the relative contributions of genetic and social determinants in this regard, just a stubborn refusal on the part of many in the human genetics community to give up on the potential of genetic discovery to contribute remedies to the problem. Having (for the most part) given up on the hope of identifying loci with reasonable effect sizes as contributing to health outcome differences, geneticists wishing to address health disparities have now turned instead to PRS.

## 4. Polygenic scores and complex disease risk

The diseases of highest public health significance, so-called ‘common complex diseases’ such as coronary artery disease, type 2 diabetes mellitus and asthma, are multifactorial in aetiology, arising from the joint influence of multiple genetic risk factors and environmental exposures. The extent of the genetic component of common disease risk varies; heritabilities, estimated from twin, adoption and studies of other relatives, of between 30% and 50% are commonly cited [31]. Whereas early theoretical work suggested that this genetic risk would be attributable to alleles present in 1–5% of the population (i.e. the Common Disease-Common Variant hypothesis [32,33]), few common variants of large effect were identified via candidate gene studies (*APOE* may be the best example of this, although its discovery also involved linkage analyses [34]). GWAS, the gene-agnostic approach which takes advantage of linkage disequilibrium across the genome to identify common variation associated with disease risk, succeeded in identifying many more significant associations (over 275 000 to date [35]), nearly all of them of individually small effect. Importantly, even when many loci have independently been associated with risk of the same disease, together they typically fail to account for more than a small portion of the expected genetic risk, as reflected in heritability estimates derived from traditional twin-studies and related methodologies (the so-called ‘missing heritability’ problem) [36,37].

One of the problems with finding risk variants using GWAS is that, to reduce the chances of spurious associations, very stringent statistical standards must be maintained. It is possible that loci that do not meet these high standards nevertheless have a measurable association with the trait in question. PRS attempt to address this problem by using many different markers, most of which do not meet standard genome-wide significance thresholds, to generate a composite measure of the overall association between those markers and the trait in question [38,39]. By lowering the statistical standards for counting a marker as trait-associated, weighting associations by estimated effect sizes and aggregating associations over a larger number of variants, predictive accuracy is increased, albeit at the expense of a loss in terms of being able to distinguish spurious from robust associations at any single locus. And, of course, any clear aetiological link between specific genetic changes and the phenotype of interest is also obscured.

Hence, while the identification of sets of loci linked to complex disease risk has helped increase confidence that fewer genetic contributions are missed, it has changed how the risk information can be translated for public health benefit. Whereas previously, the goal of most GWAS was to identify specific disease-linked genes and biological pathways with the hope of informing drug development, PRS are better suited to risk stratification not genetic discovery. PRS calculation for patients with and without the trait of interest permits estimation of the relative risk of disease for specific scores, and those at the upper limits of the population risk distribution (typically the top 2–5% [40]) can be singled out for clinical intervention. A patient who, for example, scores highly for risk of coronary heart disease could be sent for diagnostic assessment, advised to make heart-healthy lifestyle changes and/or started on pharmacological treatment, all with the intention of slowing or preventing disease processes and improving clinical outcomes (e.g. [41–43]. The primary challenge with this approach however is that, even in aggregate, effect sizes for many PRS are too small (i.e. the associations with disease risk too weak) to warrant much clinical action [44]. In other words, that someone is *slightly* more likely to at some point suffer from a complex disease such as heart disease is not necessarily useful for individual clinical (or, perhaps even, lifestyle-related) decision-making.

For phenotypes where effect sizes are higher, or other clinical risk assessments less informative, PRS could be a useful screening tool. Lello *et al.* [45] argue, for instance, that ‘the top few percentiles in PRS’ for breast cancer risk have a roughly one in three chance of developing breast cancer at some point in their lives. If correct, such individuals could appropriately be regarded as ‘high risk’ according to current guidelines (as the baseline lifetime risk is roughly 1 in 8) and therefore be worth prioritizing for earlier or more intensive cancer surveillance. The ability of PRS to improve disease risk prediction relative to other clinical risk models varies by phenotype however, even among different types of cancer [46], and many PRS only marginally improve standard clinical risk assessments [47–49].

Aside from the variable predictive power, and hence clinical utility, of PRS, there are at least two major stumbling blocks to moving PRS into clinical practice. First, PRS, in summing across many hundreds or thousands of low significance individual estimates of variant–disease association, are especially susceptible to confounding by cryptic population structure. In other words, even if stratification bias is small per locus, it can accumulate across loci in a PRS. Researchers working on GWAS generally address the difficulty of population structure by adjusting associations for a finite number of principal components of genetic variation [50]. But such statistical control is not foolproof: a few years ago, a classic GWAS on height was found to have produced spurious results due to residual genetic population structure that remained unidentified at the time of analysis [51–53]. One approach to address such confounding is to deploy methods such as ‘sib-pair controls’, or checking to see if the predictive accuracy of a particular PRS is maintained when tested within families (see box 1). When this has been done, the PRS for some traits have remained roughly as predictive as they were in the general population (e.g. body mass index); for other traits, however (e.g. educational attainment), the predictive ability has been substantially reduced [45,57,58]. At the moment there appears to be no way to tell, without running such a test, how a particular trait's PRS will perform under those conditions.

Box 1. **Sib-pair controls and population structure.**

The sib-pair method is one way to test for the problem of traits covarying with populations, and genetic differences between the populations therefore being statistically associated with the trait in question, despite not being causally involved in the development of the trait (in other words, to test for the effects of residual or cryptic population stratification). To adapt an example from Coop [54], imagine running a genome-wide association study (GWAS) on a diverse population that included both people native to, say, Liverpool, England, and people native to, say, Paris, France, and looking at the trait ‘pounds of black tea consumed per year’. Any alleles more common to people from Liverpool will be strongly associated with tea-drinking (people in Liverpool consume perhaps eight pounds of tea per year, whereas those in Paris consume less than around a half pound [55,56]). But these alleles are (presumably) not doing any causal work; rather, what matters is the sociocultural context. Nevertheless, a polygenic risk score (PRS) using these markers would be (broadly) predictive, insofar as it was able to predict who was English and hence who was more likely a heavy tea-drinker!

But now, if one tried to use the resulting PRS to predict tea-drinking behaviour within families, it would suddenly become non-predictive, as within a family, all children usually either grow up in a heavily tea-drinking household or not, and any differences between children are the result of idiosyncratic differences, and not associated with the alleles that differ in frequency between people from Liverpool and those from Paris. When the associations found are reduced radically under sib-pair control, it is usually a sign that something has gone awry (i.e. that the alleles used in the PRS are not related to the trait in question in a simple causal way).

The other problem, to which we turn in the next section, is that PRS developed for one population tend to be much less predictive when applied to other populations (however ‘population’ is understood or operationalized) [59,60]. And while there is still debate about *why* this is the case, *that* it is the case makes the clinical application of PRS fraught.

## 5. Polygenic risk scores and populations: the real problem

Most currently validated PRS have been developed using data from samples of broadly ‘European’ origin and/or ancestry [61], and because such scores do not replicate (or ‘transfer’) when applied to patients drawn from other population backgrounds, many PRS are less predictive when used for non-European ancestry populations [62]. Why this is the case remains an open question, with commentators noting the role of population genetic differences in underlying allelic architecture and patterns of linkage disequilibrium, as well as the likely contribution of non-genetic risk factors and population-specific gene–environment interactions [54,61–66] (see box 2). Irrespective of the exact explanation(s) for this, now well-recognized ‘transferability problem’, there are tangible implications of the difficulty for the deployment of PRS in clinical practice in ways that are useful and will not exacerbate health inequities. And while many, creative, solutions to the problem are currently being actively explored [63,69,70], it is not clear that even the best designed approach to PRS development and validation will be able to transcend more fundamental difficulties inherent to the distribution of health and disease among human groups.

Box 2. **Why might polygenic scores developed using one population not be predictive in other populations?**

Duncan *et al.* [61] list six potential reasons why polygenic scores developed in one population might be less predictive in other populations:

(i) True differences due to drift.

(ii) True differences due to selection.

(iii) True differences in genetic effects due to environmental differences (gene–environment interactions).

(iv) Bias due to uncorrected population stratification in discovery and/or training samples.

(v) Bias due to our discovery/training population data and/or polygenic scoring methods. Specifically, linkage disequilibrium (LD) structure and variant frequency are captured imperfectly with current methods (including genotyping and imputation), and they vary across populations, and currently available data resources are unequally representative of diverse worldwide populations.

(vi) Random error in the estimation of GWAS betas.

Both (i) and (ii) involve the possibility that differences in the genetic makeup of two populations might result in a polygenic risk score (PRS) that is predictive in one population not being predictive in the other, because of the differences in allele frequencies between the two populations (see [67] for a detailed analysis of some of the ways in which this can happen). Imagine two populations, each of which has hundreds or thousands of genes that influence a particular trait. If in the tested population, a subset of those alleles have reasonably high relative frequencies (thus permitting the detection of the effect the different alleles have), whereas in the other population, most of those loci from that same subset are fixed or nearly fixed (so these genes are not doing much ‘work’ in the population), a PRS from the first will fail to be predictive in the second, even if, in the second, those genes do ‘the same’ thing that they do in the first population.

For (iii), the issue is that an allele that, in one environment, has a positive effect on the trait (compared to the other alleles at that locus), might, in a different environment, have a much weaker effect, have no effect, or even have a negative effect, on the same trait. Here, Lewontin's ‘Analysis of Variance and Analysis of Causes’ [68] is the classic reference. One of Lewontin's arguments for analysis of variances being of limited value in understanding the causal influence of genes on traits was precisely that they revealed nothing about the ‘norm of reaction’ of the trait. He argued that understanding the norm of reaction was essential to understanding how development would respond to different environments (fig. 1 of [68]). Relatedly, a change in the environment can change the heritability of a trait [68], and some changes might reduce heritability and hence the predictive ability of PRS.

For (iv), and as per box 1, if there is population structure in the population tested, and that structure is associated with differences in traits for environmental and/or social reasons, a GWAS will be able to use the alleles that differ in frequency between the populations to predict differences in traits, despite those alleles having no true causal influence on the trait in question. In general, stratification of this sort will generate biases, and PRS, which relax the statistical requirements for using associations, are particularly sensitive to this kind of error.

For (v), recall that GWAS do not identify actual genes, but rather markers which are presumed to be associated with some (number of) gene(s). If, in one population, variation in a marker tends to be correlated with that of a particular gene (i.e. the marker and gene are in LD), but in another population LD is lower, a PRS developed using the first population will not be as predictive in the second.

Point (vi) simply notes the possibility of random errors; while this concern is considerable in GWAS, it is generally thought that markers found to be associated at the standard, very strong, statistical significance levels are unlikely to be spurious. The same cannot be said of the weaker associations used in PRS, many of which are in fact likely to be mis-estimated or entirely spurious.

Note that more than one of these problems may occur in any particular GWAS and that these different issues can interact in potentially complex ways.

More recently, Mathieson [66] has suggested that the ‘omnigenic model’ (in which many loci contribute indirectly to variation in many traits) can explain the failure of polygenic scores developed in one population to be predictive in others; he argues further that since PRS likely rely so heavily on loci with only indirect relationships to the trait in question, we should not expect them to be ‘robust clinical targets’. The mechanisms by which predictive power is lost in the omnigenic model are broadly similar to (i), (ii), (iii) and (v) described here.

For example, while the immediate source of the transferability problem is the markedly skewed (Euro-centric) underlying population distribution of global GWAS discovery efforts [59,60,71], the ascertainment and analysis of additional non-European ancestry populations is necessary but not sufficient for the clinical achievement of more generalizable risk prediction. This is because data from a more heterogeneous collection of population genetic backgrounds, where available, must still be organized prior to the development and validation of new PRS. In general, two distinct approaches are employed. Either PRS are designed to separately predict disease in each major population (or, more often, genetic ancestry) group of interest, stratifying prediction to encompass population-specific variants with stronger associations with disease risk (e.g. [72]), or an ‘all-purpose’ PRS is designed to capture and combine predictive elements from a global population sample (encompassing multiple subpopulations) into a single risk predictor that can be universally applied (e.g. [70,73]). In the former case, appropriate clinical application requires assigning the patient to a population/ancestry category prior to risk estimation; there are several difficulties with this. First, what is going to count as a ‘population’ in this context will vary based on the assumptions made by the researchers developing these tools [74]. Even leaving aside the problem of identifying ‘natural’ populations, for any set of populations developed, patients, whose ancestry is either admixed or does not otherwise correspond to the groups for which a score has been validated, may receive less accurate risk estimates. In the latter case, without a clear understanding of all of the factors that contribute to marginal effect size estimation, the PRS may still underestimate risk. This is because in the presence of gene × gene and/or gene × environment interactions, a given PRS may only predict risk for a cohort matched on both ancestry and environment.

Importantly, no matter which approach is applied, complexities of human population structure (i.e. the ways in which non-random mating occurs across different axes and scales) will stymie even good faith efforts to translate polygenic risk information for use with individual patients. First, there is no way to determine, ahead of actually testing them, which population or ancestry genetic clusters are biomedically relevant, making any choice of ancestry categories or subpopulations for effect size (and hence, PRS) estimation effectively arbitrary; a feature of human genetic variation that is well recognized [74]. Furthermore, as already noted, whatever stratification approach is adopted will fail many patients whose genetic identities do not otherwise correspond to the chosen categories.

Along with the lack of an objective way to identify populations of interest, there is the equally significant problem of the disconnect between human population genetic structure and non-genetic population arrangements such as, for example, socially ascribed racial and/or ethnic identity [75]. Often, what is medically relevant about populations (and hence what influences the predictive value of a PRS) is a function of the social and/or environmental risk factors shared by that population, and not the allele frequencies of the population in question. This fact all but ensures that ‘all-purpose’ PRS will capture spurious associations and risk that is not causally associated with the alleles in question (per box 1). And, because the impact that such structure has on health outcomes (via straightforward differences in genes causally associated with health) is likely swamped by the differences caused by social determinants of health [76,77], once again it is unclear how best to move from a population-level understanding of disease risk (however defined) to a risk prediction for an individual patient.

In other words, if the population structure that mattered for disease risk could be readily identified, and if sociocultural location mirrored genetic population structure, then population structure, understood to reflect both sociocultural position and genomic affinities, could be accounted for in the development and clinical application of PRS. Put another way, if human population structure was straightforward, i.e. if there were in fact only a few major population divisions within our species, little gene exchange between populations, and general homogeneity (of both genetic and non-genetic risk factors) within each of those populations, the problem would be easily solved. In such a world, it would be simple to develop separate PRS for each population, and easy to assign people to the correct referent population for risk analysis using a PRS. But part of the lesson from the kind of study of human populations that Lewontin made famous is precisely that this picture is utterly *unlike* actual human population structure. While it is true that sophisticated clustering algorithms can tease out elements of genetic population affinities [78–80], there is both no sense of there being a ‘lowest level’ at which population ceases to matter, nor do such algorithms produce a privileged set of clusters that are ‘right’ in all cases. Instead, there are better and worse ways of cutting the pie for particular purposes, and for the generation of PRS with clinical value, no obvious method that guarantees the capture of all and only clinically relevant risk factors.

But if developing and validating separate PRS for each population of interest is a losing proposition, there are good reasons to be sceptical that developing a PRS using a worldwide population sample could solve this problem either. Recall that predictive accuracy in populations with internal structure can be high, even if many of the variants on which the prediction is based have no causal effect whatsoever. That is, at the population level, we can successfully predict that specific alleles are associated with the trait in question, but we are doing so only because they are associated with genetic population structure, and it is the environmental differences associated with the stratified populations that are doing the real work of increasing disease risks.

## 6. Shipwrecked on the shoals of population structure

There is a certain irony in the utility of PRS being wrecked on the shoals of population structure. Twenty years ago, Rose [81] published ‘Sick Individuals and Sick Populations’, part of the point of which was to highlight the fact that the causes of differences in disease incidence *within* populations (causes of incidence) were *usually* distinct from the causes of differences in disease prevalence *between* populations (causes of prevalence). The other take-home message from Rose was that the causes of between-population prevalence were *usually* where the public health action was. If one wants to make major changes in population-level health outcomes, i.e. to identify and intervene on the risk factors responsible for health inequities, attention to the causes of prevalence usually makes more sense than a focus on identifying and intervening on individuals' exposure to the causes of incidence. But, of course, using PRS to guide clinical practice is to focus on causes of incidence (a very particular set of causes: genetic predispositions to disease), and hence to not only disregard more salient risk factors but to misdirect public health intervention.

There are at least two potential negative consequences of this misdirected attention. First, in labelling only those at the furthest limits of the incident population distribution ‘high risk’, PRS (particularly if relied on in preference to more salient, but harder to measure, social determinants of health) may contribute to false reassurance among those whose social identities nevertheless place them at higher (prevalent) risk due to the experience of systemic disadvantage and discrimination. This can promote both individual complacency and broader harms, such as those associated with delayed diagnosis and treatment. Second, it may inappropriately lay the blame for poorer health outcomes on incident (in the case of PRS, genetic or innate) susceptibilities, suggesting that the uptake of an earlier colonoscopy (or similar screening modality) by those at the highest end of the genomic risk distribution will compensate for myriad unmeasured and unaddressed social determinants of health. The degree to which these negative consequences come to pass is still an open question and, as has been argued elsewhere [43,82], empirical work exploring the impact of PRS on patients and their broader communities is urgently required lest this new approach perpetuate the very population health inequities it seeks to address.

## 7. Conclusion

The problem here, as was the case in Lewontin's intervention 50 years ago, is that neither health risks nor sociopolitical relationships can be adjudicated by scientific descriptions of human genetic variation. On its face, this is confusing: if racial discrimination is justified by supposed evidence of racial biological distinction, then surely definitive proof that such biological distinction is a fallacy would effectively, and permanently, undermine discriminatory beliefs and practices. But this ignores the complex ways in which population genetic observations are taken up and used, or alternatively ignored [83], for strategic social ends. Similarly, proponents would have us believe that attending to, and indeed accounting for, population structure in the context of polygenic risk estimation will allow us to more effectively identify and intervene on population health risks. However, because nearly all public health problems are co-constituted by biological as well as social determinants, an exclusive (or near exclusive) focus on genetic risk, once more, ignores the social realities. Only this time, much more than the apparent irrelevance of scientific claims is at stake: in the absence of a better understanding of the risk factors driving disease aetiology, disparities can only persist and even potentially worsen. This is the lesson to take away from ‘The Apportionment of Human Diversity’. Namely that science, even exquisitely executed and explained science, in the absence of social contextualization and understanding, will not solve our social ills.

## Data Availability

This article does not contain any additional data.
